# Computational modeling of spatiotemporal afterimage visual perception with spiking neural networks

**DOI:** 10.3389/fnins.2026.1780751

**Published:** 2026-03-17

**Authors:** Irena Byzalov, Hadar Cohen Duwek, Elishai Ezra Tsur

**Affiliations:** Neurobiomorphic Engineering Lab, The Open University of Israel, Raanana, Israel

**Keywords:** afterimage effects, computational modeling, perceptual filling-in, spatiotemporal dynamics, spiking neural network, visual system mechanisms

## Abstract

Contour-induced afterimages constitute an important class of chromatic visual illusions, in which an illusory color percept emerges post-exposure to a chromatic field. Their striking feature is dual polarity (the perception of both complementary and inducer hues) and the capacity for extending to naive, non-adapted regions, indicating the involvement of neural mechanisms that extend beyond established models of simple neural adaptation. In this work, we realized the perceptual afterimage effect with a biologically plausible spiking neural network. We compared the results with experimental findings with human participants, demonstrating how a complex temporal evolution of a visual illusion can emerge from the dynamics of its constituent spiking dynamics. Our neural design models a wide range of phenomena, including positive, negative, and combined afterimage configurations, as well as the effects of alternating and open contours. By intrinsically incorporating the temporal dimension through its spiking dynamics, the model accurately reproduces the temporal evolution of the perceived color, including the alternating polarity observed with successive contours. We show that a single, unified, and biologically plausible spiking architecture can account for both veridical color and the complex set of contour-induced afterimage phenomena, suggesting that a common, active neural process, chromatic filling-in, is responsible for the different forms of perceived color. From an engineering perspective, our model exemplifies neuromorphic computational processing of event-based representations of visual data without reducing to static frames, and enables systematic analysis of inference error and illusory afterimages through configurable parameters, offering conceptual guidance for designing bio-inspired neuromorphic imaging pipelines.

## 1 Introduction

**Figure 1:**
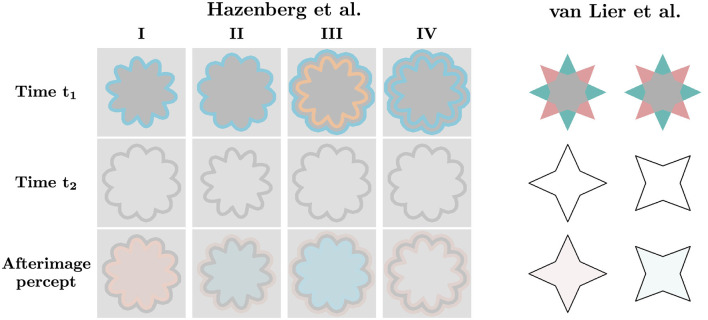
Illustration of contour-induced afterimage filling-in effects. Contour stimuli based on Hazenberg and van Lier (2013) are shown on the **(left)**, and star stimuli based on van Lier and Vergeer (2009) on the **(right)**. The first and second rows show the initial chromatic stimulus and the subsequent achromatic test contour, respectively, and the last row illustrates the perceived image. The contour stimuli are presented in four configurations: I. Negative effect induced by a contour outside the chromatic stimulus. II. Positive effect induced by a contour inside the chromatic stimulus. III. Constructive interaction between positive and negative effects induced by contours of opposite colors produces a stronger filling-in effect. IV. Destructive interaction between positive and negative effects induced by contours of the same color cancels the filling-in. Afterimages adjacent to the contour may remain imperceptible or emerge after longer exposure. In the star stimuli, an achromatic contour coinciding with one of the stars induces uniform filling-in of the color complementary to that star within the contour.

Visual illusions have long been instrumental in investigating the neural processes underlying visual perception. One of the most familiar examples is the color afterimage: prolonged fixation on a colored stimulus elicits the perception of a complementary color once the stimulus is replaced with a blank background. In the classical afterimage effect, negative afterimages appear in the regions directly stimulated by color. However, multiple studies have shown that afterimage perception can be modulated by luminance contrast; for instance, afterimages appear stronger when surrounded by luminance edges (Daw, 1962; Powell et al., 2012). Contours can induce illusory color filling-in aftereffect also in regions not directly exposed to color, and with hues not limited to the complementary of the inducer. Such illusory filling-in can be elicited both by chromatic contours concurrently with their presentation, as in the watercolor effect Pinna et al., (2001); Pinna, (2006), and by achromatic contours presented after an initial chromatic stimulus (Anstis et al., 1978; van Lier and Vergeer, 2009; Hazenberg and van Lier, 2013; Barkan and Spitzer, 2017). For example, Anstis et al. (1978) demonstrated a positive afterimage: following adaptation to a colored field, the gray region it enclosed appeared to take on the inducer's hue. The authors attributed this to a combination of simultaneous contrast induction and adaptation. In simultaneous contrast induction, the perceived chromaticity of a region is shifted toward the complementary hue of its surround, a process thought to be mediated by lateral inhibition (King and Wertheimer, 1963; Shively, 1973).

van Lier and Vergeer (2009) demonstrated that achromatic test contours can elicit distinct afterimages from the same chromatic stimulus. Their stimulus consisted of two overlapping quadrilateral stars with complementary colored spikes and a gray central overlap (Figure 1, right column). An achromatic contour outlining one of the stars induced a complementary hue of that star within its boundary, including in the overlapping area that had not been exposed to color. The authors showed that the outlined star's chromatic spikes induced a complementary hue inside the contour, whereas the second star's spikes outside the contour induced the same hue inside the contour, albeit more weakly. When the two contours were presented in succession, the perceived afterimage color alternated accordingly. Another example of contour-dependent percepts arising from the same chromatic stimulus was demonstrated (Anstis et al., 2012). The authors presented multicolored stimuli followed by a test contour enclosing several regions of different colors. Within a given contour, the colors blended into an averaged afterimage, whereas the colors remained distinct across contours.

Hazenberg and colleagues further investigated afterimage filling-in using thin closed chromatic contours, with an achromatic contour positioned either inside or outside the chromatic stimulus (Hazenberg and van Lier, 2013). The direction of filling-in depended on this spatial relationship: an outer achromatic contour induced a complementary color within its bounds (negative effect), whereas an inner achromatic contour induced the same color as the adapting contour (positive effect). Additionally, they showed that multiple chromatic contours can be combined to produce a cumulative filling-in effect. When a pair of chromatic contours flanked the subsequent achromatic contour, the perceived color was enhanced if the chromatic contours were complementary, and canceled if they were the same color. Alternating positive and negative effects could also be induced by presenting outer and inner achromatic contours in succession. Figure 1, left column, illustrates the negative, positive, and the two double-contour configurations. In these experiments, the chromatic stimulus and the achromatic contour were each presented for one second in a cyclic sequence. The chromatic stimulus included a gray inner region matched in luminance to the colored contour; however, iso-luminance is not a prerequisite for the effect to occur, though it may affect its strength.

Although adaptation is presumed to be involved in contour-induced afterimages, their characteristics differ markedly from those of classical afterimages. In addition to exhibiting dual polarity and spreading into non-adapted regions, contour-induced afterimages emerge after much shorter exposure time and can be triggered even by a thin chromatic contour, whereas classical afterimages typically require relatively long adaptation and large chromatic areas sufficient to counteract involuntary saccadic motion. Additional neural processes, beyond adaptation, must therefore be involved, yet their nature remains unknown. This is unsurprising, given that even the neural mechanism of classical afterimages remains under debate. Proposed mechanisms range from photopigment bleaching in the retina to cortical adaptation (Williams and Macleod, 1979; Shimojo et al., 2001; St.Clair et al., 2007; Zaidi et al., 2012; Webster, 2015; Zeki et al., 2017), where multiple mechanisms may act in concert or under different conditions. For example, Zaidi and colleagues demonstrated that under normal light conditions, adaptation is slower than would be expected from photopigment bleaching (Zaidi et al., 2012). Instead, they attributed afterimages to the rebound response of inhibited retinal neurons, which fire bursts once the inhibition is released (Spitzer et al., 1993).

The neural basis of perceptual filling-in remains uncertain. Two prominent theories have been proposed: (i) the isomorphic theory, which postulates that neural signals spread from edges across the retinotopic map to reconstruct a two-dimensional representation of the visual field, and (ii) the symbolic (or cognitive) theory, which holds that shapes and colors are represented at higher levels of visual processing without requiring an explicit spatial representation (Cohen-Duwek and Tsur, 2021). Both theories have experimental support (Komatsu, 2006; von der Heydt et al., 2003).

Computational models attempting to predict these effects are scarce, all following the isomorphic theory. For example, the FACADE model (Grossberg and Mingolla, 1985) and its later variants (Grossberg and Todorovic, 1988; Francis and Ericson, 2004; Francis, 2010) have been used to predict various afterimage effects, but could not account for positive afterimages. Cohen-Duwek and Spitzer (2018) developed a compound computational model that successfully accounted for both positive and negative afterimages through a single mechanism, modeled as a diffusion process with boundary conditions defined by the reversed chromatic gradients of the initial stimulus, amplified at locations coinciding with the achromatic test contour. This amplification was implemented by multiplication with the test contour, an operation that has no immediate counterpart in neural circuitry. In their model, all chromatic edges contributed cumulatively; however, the amplified edges ultimately determined the polarity of perceptual filling-in.

In this work, we extend this approach with a biologically inspired spiking implementation. Spiking neural networks (SNNs) provide a framework for simulation of high-level perceptual phenomena grounded in biologically plausible modeling of low-level neural activity (Tsur, 2021). SNNs were used to model various visual illusions, including color constancy, color assimilation, ambiguous color perception (Cohen-Duwek et al., 2022), and illusionary contrast perception (Cohen-Duwek and Tsur, 2022).

SNNs inherently model the temporal, event-driven nature of neural signaling. Our model captures the unfolding spatiotemporal dynamics of early visual processing. It spans the entire temporal sequence of the experiment and, crucially, is self-contained: its predictions arise directly from its intrinsic state, driven solely by the time-varying stimuli. These properties allow the model to generate predictions that trace the temporal progression of perception within and across experimental stages, while also supporting the simulation of more complex temporal effects, such as three-stage alternating contours.

## 2 Methods

**Figure 2:**
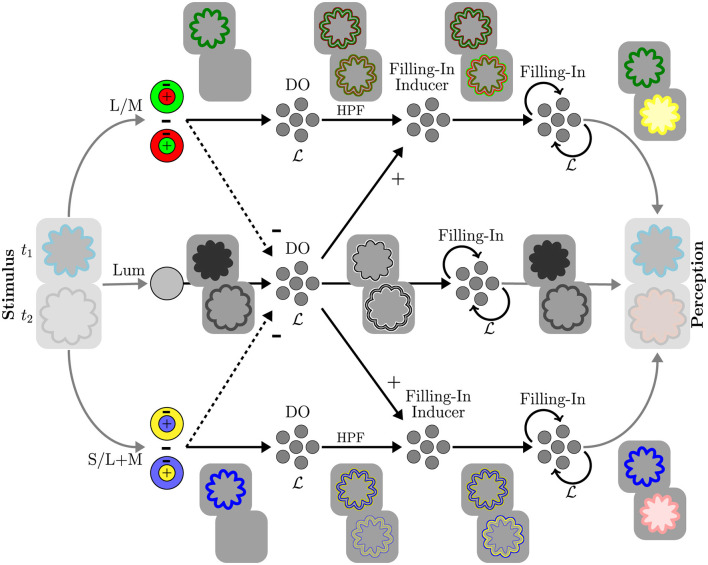
SNN architecture and illustrative intermediate representations. DO: double opponency; HPF: high-pass filter; L: discrete Laplacian; (+) denotes amplification via excitation; (–) denotes inhibition.

Our model was implemented as a spiking neural network (SNN), designed to simulate key functional components of the early visual system and to intrinsically capture both spatial structure and temporal dynamics. An overview of the model architecture is shown in Figure 2. Visual stimuli are encoded into three opponent channels: red/green, blue/yellow, and luminance. These channels reflect the single-opponent organization of retinal ganglion cells and the lateral geniculate nucleus. These opponent channels are processed along separate functional pathways. Single-opponent signals feed into the double-opponent edge-detection component, corresponding to double-opponent neurons in the primary visual cortex that encode visual information as chromatic and spatial boundaries. Chromatic double-opponent signals undergo neural adaptation, and the resulting adapted signals are subsequently modulated by the luminance channel's double-opponent output, which enhances chromatic gradients at the test contour. Each pathway implements diffusion-based filling-in, following the isomorphic theory of perceptual filling-in. The filling-in is driven by modulated edges in the chromatic channels and by luminance edges in the luminance channel. The outputs of all channels are then combined and integrated to yield the final percept. Subsequent sections describe the components in detail.

### 2.1 Neural engineering framework

Our SNN model is implemented using the Neural Engineering Framework (NEF) (Eliasmith and Anderson, 2003; Tsur, 2021), a theoretical and computational framework for building large-scale, biologically plausible neural models. In NEF, information is represented by the activity of neural populations through nonlinear encoding, and is recovered or transformed via weighted linear decoding.

The neural activity *a*_*i*_ of neuron *i*, encoding an input vector *x*, is given by:


$$\begin{array}{l} a_{i}=G_{i}\left[\alpha_{i}e_{i}\cdot x+J_{i}^{\text{bias}}\right] \end{array}$$ (1)

where *G*_*i*_ is the neuron model, *e*_*i*_ is the neuron's preferred direction (encoder), α_*i*_ is a gain factor, and $J_{i}^{\text{bias}}$ is a fixed background current. *G*_*i*_ may be any neuron model that maps input current to activity. In spiking neuron models such as leaky integrate-and-fire (LIF), activity is expressed as a spike train.

This distributed population encoding can be decoded as:


$$\begin{array}{l} \hat{x}=\overset{N}{\underset{i=1}{\sum }}a_{i}d_{i} \end{array}$$ (2)

where *N* is the number of neurons in the population, and *d*_*i*_ is the decoder vector for neuron *i*, derived by solving a reconstruction problem via least-squares minimization.

Neuronal ensembles communicate through weighted synaptic connections. An arbitrary transformation *f*(*x*) can be implemented by optimizing the decoders to approximate *f*(*x*) instead of *x*.

Although decoding is abstractly framed as a reconstruction process, this formulation is equivalent to embedding the decoders within the synaptic connection weights, computed as the outer product of the decoders and the encoders of the downstream neurons. NEF acts as a neural compiler: given a neuron model, its parameters, and the target values and computations, it optimizes the synaptic weights accordingly.

NEF models biological synapses as filtering operations, with the low-pass filter serving as the default model.

Under this synaptic model, NEF provides a method for implementing dynamical systems of the form:


$$\begin{array}{l} \frac{dx}{dt}=f\left(x\left(t\right)\right)+g\left(u\left(t\right)\right) \end{array}$$ (3)

where *x* is a state variable represented by the neural activity of an ensemble, *u* is the input, and *f* and *g* are arbitrary functions. This dynamics can be implemented by setting the input connection to compute the function


$$\begin{array}{l} ĝ\left(u\right)=\tau g\left(u\right) \end{array}$$ (4)

and the recurrent connection to compute the function


$$\begin{array}{l} \hat{f}\left(x\right)=\tau f\left(x\right)+x \end{array}$$ (5)

where τ is the time constant of the low-pass synaptic filter of the recurrent connection.

### 2.2 Model inputs

The input RGB image, representing the visual stimulus, is converted into an opponent-color space comprising two chromatic opponent channels, L/M and S/(L+M), and one achromatic channel, following established biological pathways. The L/M channel captures the opponency between long-wavelength (red) and medium-wavelength (green) light; the S/(L+M) channel represents the opponency between short-wavelength (blue) and the combined response to long- and medium-wavelengths (yellow); and the achromatic channel reflects overall luminance (Kandel et al., 2021).

We approximate the conversion of the input RGB image into an opponent-color space by the linear transformation *M* (van de Sande et al., 2010):


$$\begin{array}{l} \left(\begin{array}{c} SO^{\left(RG\right)}\\ SO^{\left(BY\right)}\\ Lum \end{array}\right)=M\left(\begin{array}{c} R\\ G\\ B \end{array}\right)=\left(\begin{array}{ccc} \frac{1}{\sqrt{2}} & -\frac{1}{\sqrt{2}} & 0\\ -\frac{1}{\sqrt{6}} & -\frac{1}{\sqrt{6}} & \frac{2}{\sqrt{6}}\\ \frac{1}{\sqrt{3}} & \frac{1}{\sqrt{3}} & \frac{1}{\sqrt{3}} \end{array}\right)\left(\begin{array}{c} R\\ G\\ B \end{array}\right) \end{array}$$ (6)

where *SO*^(*RG*)^, *SO*^(*BY*)^ and *Lum* correspond to the single-opponent channels L/M and S/(L+M), and the luminance channel, respectively. The three resulting channels serve as inputs to the spiking neural network.

### 2.3 Double-opponency and adaptation

The first stage in each chromatic pathway performs edge detection on the corresponding opponent input using a second-order Laplacian filter. This stage models the behavior of double-opponent cells in V1, which are sensitive to oriented chromatic edges (Shapley and Hawken, 2011; Shapley et al., 2019; McIlhagga and Mullen, 2018). In practice, edge detection is implemented as a convolution with the discrete Laplacian kernel L:


$$\begin{array}{l} DO^{\left(ch\right)}=SO^{\left(ch\right)}*\left(k_{ch}L\right),\;L=\left(\begin{array}{ccc} 0 & 1 & 0\\ 1 & -4 & 1\\ 0 & 1 & 0 \end{array}\right) \end{array}$$ (7)

where *SO*^(*ch*)^ is the signal on the input channel *ch*∈*RG, BY, Lum* in the opponent space, and *k*_*ch*_ is a scaling constant that can be configured per channel for flexibility, and * denotes convolution. The scaling serves to amplify the input for signal stability and carries no biological interpretation.

The detected spatial edges are subject to neural adaptation. To a first approximation, the decay in neural response to a sustained stimulus is exponential, with low-frequency components being suppressed—a fingerprint of a high-pass filter (HPF) (Benda, 2021). In particular, Zaidi et al. (2012) showed that electrophysiological recordings during afterimage-inducing stimulation were well fit by an adaptive model subtracting an accumulating, exponentially decaying signal, which is also equivalent to HPF. Following this approach, we model the adapted edges as:


$$\begin{array}{l} DO_{ada}^{\left(ch\right)}=DO^{\left(ch\right)}*HPF \end{array}$$ (8)

Concretely, we replace the NEF default low-pass synapse with a high-pass synaptic model on the outgoing connections of double-opponent ensembles. Upon stimulus offset, when the chromatic contour is replaced by an achromatic one, the adapted neurons produce reversed chromatic edges, consistent with the rebound response proposed by Zaidi et al. (2012) as a neural basis of negative afterimages.

Our synaptic model can be viewed as an abstraction of synaptic depression, and its placement in the network corresponds to cortical rather than retinal adaptation, as argued by Zaidi et al. (2012). While synaptic depression is one possible mechanism of adaptation, and cortical adaptation may contribute to contour-induced afterimages, which, as discussed previously, cannot be explained solely by the adaptation process, our implementation is intended primarily as a simple model that captures the effect of adaptation dynamics on chromatic gradients. The specific implementation and the choice to apply it after the double-opponent stage rather than before are not crucial for reproducing the effect.

### 2.4 Chromatic filling-in

Following Cohen-Duwek and Spitzer (2018), we formulate the filling-in as a diffusion process and assume that afterimage filling-in is triggered by adapted chromatic edges, modulated by the achromatic contour. Our model realizes the perceptual filling-in of both veridical and illusory colors through a unified mechanism, made possible by the use of a high-pass filter, which generates the rebound response at stimulus offset. Both edge extraction and modulation are performed intrinsically within the model. The following subsections describe (i) the mechanism for edge-driven amplification of the chromatic inducer and (ii) the diffusion-based process.

#### 2.4.1 Chromatic inducer

**Figure 3:**
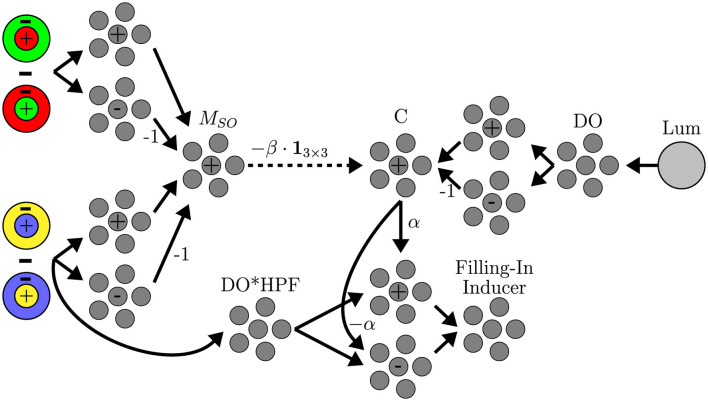
Chromatic gradient modulation mechanism. +, – denote ensembles tuned to positive and negative values, respectively, that pass those values through unchanged. The values on connections represent linear transformations. C: the modulatory signal; M_*SO*_: combined chromatic single-opponent magnitude; α, β: amplification and inhibition factors; **1**_3 × 3_: box filter. DO, HPF, Filling-in Inducer correspond to those in Figure 2. L/M channel modulation (not shown) is analogous to the depicted S/L+M channel.

The adapted chromatic gradients are modulated by an additive signal, driven by an excitatory input from the luminance double-opponent response and gated by an inhibitory input from the single-opponent chromatic response. Figure 3 illustrates the modulation mechanism. The modulatory signal enhances chromatic gradients that spatially coincide with the achromatic contour. When a chromatic stimulus is physically present, it delivers a strong inhibitory input that suppresses the modulation. This behavior arises naturally from processes that operate locally and uniformly across all pixels.

The excitatory input is provided by the luminance double-opponent magnitude mask. The double-opponent response includes both positive and negative values, corresponding to opposite edge polarities. To compute its magnitude, the signal is projected through two neural ensembles: one responsive to positive values and the other to negative values. These outputs are then combined to produce the luminance edge magnitude signal |*DO*^(*Lum*)^|.

The inhibitory signal is derived from additively combined single-opponent magnitude masks of the two chromatic channels. Magnitudes are computed in the same manner as for the luminance edge and then combined into a single mask


$$\begin{array}{l} M_{SO}=|SO^{\left(RG\right)}|+|SO^{\left(BY\right)}| \end{array}$$ (9)

Since edges span both sides of a boundary, the single-opponent response is spatially dilated using a simple 3 × 3 box filter, denoted **1**_3 × 3_, to ensure sufficient overlap with the edge region. Finally, the signal is scaled by an inhibition factor β, chosen large enough to suppress modulation in the presence of a single-opponent chromatic response (in practice, dilation and scaling are folded into a single transform). The modulatory signal *C* is represented by a neural ensemble selective to positive values. The described neural computation is approximately equivalent to:


$$\begin{array}{l} C=\max\left(|DO^{\left(Lum\right)}|-\beta M_{SO}*1_{3\times 3},0\right) \end{array}$$ (10)

The adapted chromatic gradient is decomposed into positive and negative components. The modulatory signal, scaled by the amplification factor α, is applied to each with the matching sign, and the results are then recombined to yield the final filling-in inducer *E*^(*ch*)^. This can be expressed as:


$$\begin{array}{l} E^{\left(ch\right)}=\mathrm{sign}\left(DO_{ada}^{\left(ch\right)}\right)\cdot \left(|DO_{ada}^{\left(ch\right)}|+\alpha C\right); \end{array}$$ (11)

Note that while negative values are not physiologically realistic, in our model, they simply represent neural responses to stimuli of opposite polarity. This allows the system to distinguish between opposing conditions, such as edge direction or color opponency, while still relying on biologically plausible population activity to encode the neural response.

#### 2.4.2 Diffusion-based filling-in

Following our earlier work (Cohen-Duwek et al., 2022), we realized a recurrent spiking neural network to reconstruct the perceived color from chromatic edges, based on the following diffusion equation:


$$\begin{array}{l} \frac{\partial I}{\partial t}=c_{r}\Delta I\left(x,y\right)+c_{i}\nabla \cdot \left(\nabla I_{\text{in}}\left(x,y\right)\right) \end{array}$$ (12)

where *I* is the reconstructed image, *I*_in_ is the stimulus, Δ is the Laplacian operator, ∇· is the divergence operator, ∇ is the gradient operator, and *c*_*r*_ and *c*_*i*_ are the diffusion coefficients.

The term ∇·(∇*I*_in_(*x, y*)), equal to Δ*I*_*in*_(*x, y*), corresponds to the edge structure of the stimulus being reconstructed. In our network, the diffusion process operates on the chromatic inducer *E*(*x, y*) (Equation 11):


$$\begin{array}{l} \frac{\partial I}{\partial t}=c_{r}\Delta I\left(x,y\right)+c_{i}E\left(x,y\right) \end{array}$$ (13)

As was described above, the inducer automatically captures both the chromatic gradients of physically present stimulus and the adapted gradients that arise after its offset, which respectively drive the reconstruction of veridical color and the generation of illusory afterimages within a single diffusion process.

The diffusion process described by Equation 13 realizes the general dynamics of Equation 3 and can therefore be implemented following Equations 4–5. Concretely, it is implemented using a recurrent connection with time constant τ_*r*_, which computes $\tau_{r}c_{r}L\left(I\left(x,y\right)\right)+I\left(x,y\right)$, where L denotes a discrete Laplacian, and an input connection that computes τ_*r*_*c*_*i*_*E*(*x, y*). Each neuron is connected to its four immediate neighbors through the recurrent connections, realizing horizontal neural connections.

### 2.5 Luminance filling-in

The luminance output was reconstructed by edge-driven diffusion, implemented as a recurrent connection, similarly to the chromatic channels. Luminance edges, extracted once using a Laplacian filter, served both to trigger filling-in within the luminance channel and to modulate the chromatic channels.

### 2.6 Perceived image

The spiking network generates dynamic outputs over the course of the simulation. First, we temporally sample the chromatic channels outputs, after filling-in, at different time points using a low-pass probe. When a single-opponent chromatic signal is present, we use it as an anchor to rescale the chromatic outputs back to their original range through a simple affine transformation. Rescaling is performed independently per chromatic channel and per sampled frame. Luminance output is scaled in a similar manner.

Finally, we generate the RGB image representing the model's predicted percept. The rescaled chromatic channels and the luminance channel are combined and converted to RGB using the inverse opponent-color transformation:


$$\begin{array}{l} \left(\begin{array}{c} R\\ G\\ B \end{array}\right)=M^{-1}\left(\begin{array}{c} RG_{out}\\ BY_{out}\\ Lum_{out} \end{array}\right) \end{array}$$ (14)

where *RG*_*out*_, *BY*_*out*_, and *Lum*_*out*_ are the rescaled outputs of the RG, BY, and Lum processing channels, respectively, and *M* is the opponent-color transformation from Equation 6.

## 3 Results

### 3.1 Simulation details

The spiking neural network was implemented in Bekolay et al. (2014), a Python library for large-scale neural modeling based on NEF. Spiking rectified linear neurons were used throughout, except in the diffusion module, which employed leaky integrate-and-fire (LIF) neurons. Each rectified linear neuron responded to a single polarity; thus, opposing conditions were encoded with two neurons per pixel, and single-polarity inputs with one. The parameters for the rectified linear neurons were: maximal firing rate 200 Hz, encoders 1 and –1 for the opposite polarities, and intercept 0, except for the magnitude mask component, where the intercept was set to 0.1. The diffusion modules used 200 LIF neurons per pixel, with default Nengo parameters (RC time constant 0.02 s and refractory period 0.002 s). Unless stated otherwise, default Nengo synapses were used (low-pass with time constant 0.005 s). Spatial filtering was applied using Nengo's convolutional transform, with one-pixel padding on each side: edge padding in the double-opponent module and zero padding in the diffusion module.

The model's hyperparameters were tuned using the stimuli shown in Figure 1. Simulations with other stimuli used the same parameters unless stated otherwise. For edge detection, parameters were set to *k*_*RG*_ = *k*_*BY*_ = 5 and *k*_*Lum*_ = 2 (Equation 7). The time constant of the chromatic adaptation high-pass filter was set to 1 s. Diffusion coefficients were *c*_*r*_ = 2 and *c*_*i*_ = 0.25, with a recurrent time constant τ_*r*_ = 0.01*s* (Equation 12). For the inducer signal parameters were: amplification factor α = 10, and single-opponent inhibition factor β = 100 (Equation 10). Constant factors in convolution operations were folded into the kernels.

Simulations were accelerated using the Nengo OpenCL-based simulator (Bekolay et al., 2014).

### 3.2 Model predictions and analysis

#### 3.2.1 Closed contours stimuli

**Figure 4:**
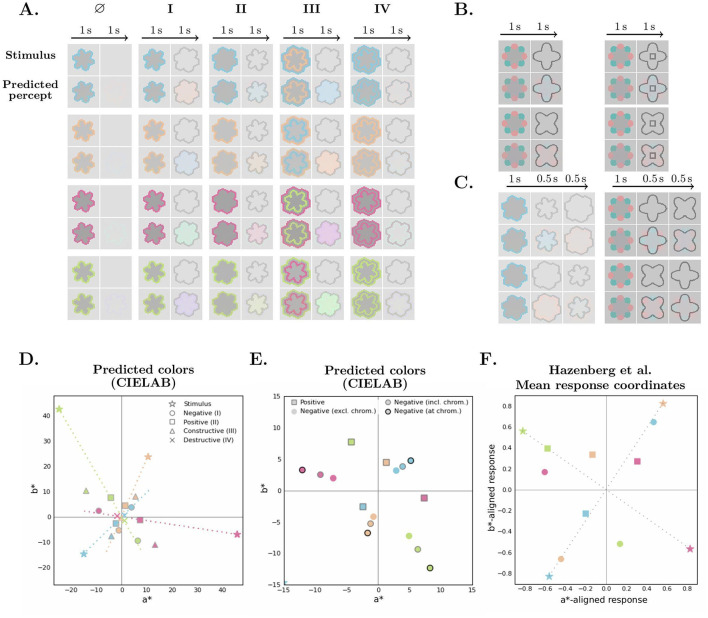
Simulation results across different configurations. **(A–C)** Show the stimuli and corresponding predicted percepts. Each simulation result is presented as a two-row panel: the top row shows the input stimuli, and the bottom row shows the predicted percepts. Columns reflect the temporal progression of stimulus presentation, with durations indicated above each column and predictions sampled at the end of each stage. **(A)** Stimuli based on Hazenberg and van Lier (2013) in four colors across five conditions: the baseline configuration ∅ (no contour) and configurations I–IV corresponding to those in Figure 1. **(B)** Star-like stimuli (left column), and the same stimuli with an added inner achromatic contour (right column). **(C)** Three-stage configurations: a chromatic stimulus is followed by two sequential achromatic contours. **(D)** Predicted percepts from A plotted in CIELAB color space. Marker color matches the chromatic stimulus (for constructive combination, the outer contour color). **(E, F)** Qualitative comparison to Hazenberg and van Lier (2013) for positive and negative conditions; **(E)** Predicted colors, with the negative mean color computed in three ways: inside the test contour (either including or excluding the chromatic region of the initial stimulus), and over that chromatic region only. **(F)** Mean response coordinates reproduced from Hazenberg and van Lier (2013), with axes (orange–blue, pink–green) rotated to approximately align with stimulus orientations in CIELAB a*-b* plane.

We first evaluated our model using stimuli that closely followed the specifications of Hazenberg and van Lier (2013), namely a chromatic contour on a gray background, with isoluminant interiors, tested across four colors and the four contour configurations presented in Figure 1. The original xyY values and the corresponding sRGB values used in our simulations are provided in Table 1. Additionally, we simulated a null condition in which the chromatic stimulus was followed directly by the background, without a contour. As in the original study, the chromatic and achromatic stimuli were presented for 1 second each. The simulation inputs were 36 × 36 pixels in size, and the width of the contours was 2 pixels. Figure 4A shows the stimuli and the predicted percepts, sampled at times 1 and 2 seconds, respectively.

**Table 1:** Stimuli colors in the CIE xyY color space and the corresponding sRGB values, obtained through a standard transformation.

**Color**	**CIE xyY**	**sRGB**
Green	(0.3514, 0.4417), 65.69	(193, 223, 129)
Orange	(0.3774, 0.3694), 58.65	(236, 193, 157)
Blue	(0.2600, 0.3058), 52.90	(142, 202, 219)
Pink	(0.3814, 0.2733), 29.87	(218, 115, 162)
Background	(0.3128, 0.3303), 73.87	(223, 223, 223)
After-contour	(0.3128, 0.3303), 55	(196, 196, 196)
Red star	(0.3768, 0.3285), 41.91	(221, 156, 157)
Cyan star	(0.2597, 0.333), 40.95	(109, 184, 181)
Overlap	(0.3127, 0.329), 42.87	(175, 175, 175)
Background	(0.3127, 0.329), 58.13	(200, 200, 200)

Contours stimuli after Hazenberg and van Lier (2013) (upper section) and stars stimuli after van Lier and Vergeer (2009) (lower section).

Figure 4D shows the predicted colors, averaged over the interior of the achromatic contour, plotted in the a^*^–b^*^ plane of the CIELAB color space. This space approximates perceptual uniformity, with the a^*^ and b^*^ axes representing the red–green and yellow–blue chromatic dimensions, respectively. In polar coordinates, the angle and the radius represent hue and chroma, respectively. The predicted colors correspond to the same hue in the positive condition and to the opposite hue in the negative condition, with lower chroma in both cases, consistent with Hazenberg and van Lier (2013). The blue and orange stimuli are sufficiently close to complementary in the CIELAB space, so the positive percept of one closely resembles the negative percept of the other. In contrast, the pink and green stimuli are not well aligned in the CIELAB space, and their percepts appear clearly distinct (Figure 4A). These results are in agreement with our perceptual observations.

In the destructive combination condition, the predicted colors are located near the origin, as expected. In the constructive combination, the predicted color exhibits a higher chroma than in the positive or negative condition alone. These results are consistent with Hazenberg and van Lier (2013). The predicted color coordinates are approximately a vector sum of the outer contour's positive and the inner contour's negative predictions.

We qualitatively compared our model predictions (Figure 4E) with the mean response analysis performed by Hazenberg and van Lier (2013) (Figure 4F) for the positive and negative conditions. In their experiment, the participants selected which of the four stimulus colors or the background best matched the perceived color. The resulting mean response diagram shows the proportion of responses for each color, plotted in a plane defined by the pink–green and blue–orange axes. We reproduced this diagram and rotated it to approximately align with the corresponding color directions in CIELAB space. Although the comparison is limited, both representations reveal similar general trends. For instance, both present closer alignment between percepts for opposite conditions of blue and orange stimuli than those of pink and green, reflecting their relative position in the color space.

**Figure 5:**
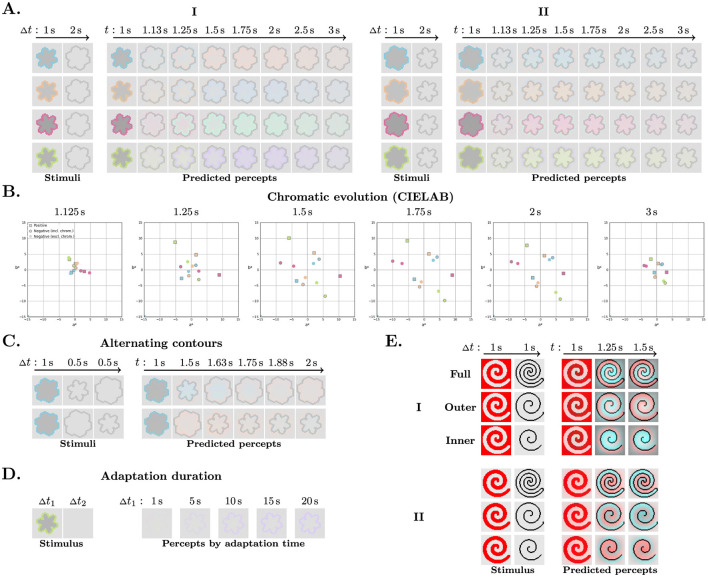
Temporal dynamics **(A–D)** and the spiral stimulus **(E)**. **(A)** Predicted temporal progression of contour-induced afterimage filling-in for the negative (left) and positive (right) conditions. **(B)** Chromatic evolution of predicted percepts from A in the CIELAB a*–b* plane. **(C)** Temporal progression under the alternating-contours condition. **(D)** Effect of adaptation duration on afterimage strength in the null condition. Top row presents the stimuli; lower rows present predicted percepts from runs with different chromatic-stimulus durations. **(E)** Spiral stimulus with three different test contours. Two chromatic stimulus configurations are shown: I. a light spiral on a red background; II. a red spiral on a light background. In each case, the same chromatic stimulus (first column on the left) is followed by one of three contour variations: full contour (first row), outer edge of the contour (second row), and inner edge of the contour (third row). The corresponding predicted percepts are shown to the right.

We further analyzed the temporal dynamics of our model, presented in Figures 5A, B. The model parameters were tuned to ensure a clearly visible color at the end of the test stage, using the same timings as in the original experiment, with no explicit constraints on the dynamics. The model's dynamics arise from the interaction of two processes: filling-in, implemented as an iterative diffusion that generates perceived colored surfaces, and double-opponent adaptation. Adaptation exerts dual and opposing effects: on one hand, stronger adaptation enhances chromatic gradients and drives more pronounced filling-in; on the other hand, it accelerates the fading of color. Together, these effects produce an initial build-up of chroma that then remains steady for most of the test stage before decaying rapidly. The temporal profile is broadly consistent with perceptual observations: the illusory color typically fades within seconds. However, the percept itself seems to appear instantaneously, whereas in the model, the build-up is slow and gradual. In the negative condition, there is an additional delay between filling-in at the adapted region and in the remaining interior. This can be attributed to the weaker inner chromatic edge (not amplified by the test contour), which drives diffusion in the opposite direction, hindering its spread toward the center.

#### 3.2.2 Star-like stimuli

We also evaluated the model using stimuli based on the star-shaped configurations introduced by van Lier and Vergeer (2009). Our stimuli were conceptually identical, with the only modification being the rounding of the stars' spikes to produce a smoother appearance in the low-resolution simulation input image. We followed the original color specifications from van Lier and Vergeer (2009), provided in Table 1, along with the corresponding sRGB values used in our simulations. The simulation inputs were 50 × 50 pixels in size, and the width of the contours was 2 pixels.

Simulation results are shown in Figure 4B. The left column displays the star-like stimuli, and the right column presents the same stimulus with an additional inner achromatic contour, as introduced by Francis (2010). Our model correctly predicts the negative afterimage corresponding to the shape enclosed by the achromatic contour, with smooth filling-in of the overlapping achromatic area. The additional inner contour, which does not coincide with a chromatic edge, does not block the filling-in, consistent with psychophysical evidence (Kim and Francis, 2011).

#### 3.2.3 Alternating achromatic contours

Next, we evaluated the model's dynamic behavior using a three-stage stimulus, in which a single chromatic stimulus was followed by two alternating achromatic contours (Figure 4C). In the first configuration, a chromatic contour was followed either by an inner contour and then an outer contour (top left), or by an outer contour and then an inner contour (top right), producing alternating positive and negative effects. We also examined the star-like stimuli, in which the alternating contours corresponded to one of the overlapping shapes in the initial chromatic stimulus. The model reproduced all observed outcomes, indicating that its internal state retained sufficient chromatic and spatial information to generate the appropriate response without re-presentation of the chromatic stimulus. Figure 5C shows the temporal progression of the predicted percepts.

#### 3.2.4 Spiral stimuli

We further tested our model on spiral stimuli based on stimuli used by Cohen-Duwek and Spitzer (2018) to test the effect of open contours. The initial chromatic stimulus features a white spiral on a red background. The achromatic test contour can be one of three variations: a full contour, its outer edge, or its inner edge. The RGB values were (250, 0, 0) for the red background, (230, 230, 230) for the spiral and the background of the test contours, and (10, 10, 10) for the test contours. The simulation inputs were 50 × 50 pixels in size, and the width of the contours was 2 pixels. Figure 5E shows the stimuli and our model's predictions. Positive effect dominated when only the outer edge was presented, while a dominantly negative effect was induced by the inner edge. With the full contour, there was a clear separation between the positive effect inside the spiral and the negative outside. These results are consistent with Cohen-Duwek and Spitzer (2018). In addition, we successfully replicated all three simulations with inverted chromatic stimulus colors, i.e. a red spiral on a light background.

## 4 Discussion

In this work, we propose a biologically plausible spiking neural model that implements and integrates core components of the early visual system. Our model successfully predicts contour-induced positive, negative, and combined afterimages, as well as veridical color perception, through a single mechanism. It also reproduces classical afterimages and the effect of adaptation duration on their strength (Figure 5D). These results suggest that filling-in across all of these cases may be mediated by a common neural process.

While the model of Cohen-Duwek and Spitzer (2018) could predict positive and negative afterimages, our model offers an important extension. Beyond its biological plausibility, the spiking formulation naturally incorporates the temporal dimension. Our model spans all phases of the experiment: inputs are received as a continuous temporal sequence that mirrors the timing of stimuli presented to observers, and predictions are generated as a matching temporal sequence. The spatiotemporal dynamics and all intermediate states are encoded intrinsically through the spiking representation, driven solely by the inputs. In particular, our model introduces an adaptation component and implements a dynamic, rather than static, diffusion process. Consequently, it enables (i) simulation and testing of multi-stage spatiotemporal effects and (ii) evaluation of temporal dynamics in addition to end-point percepts. Our model follows earlier attempts in amplifying chromatic gradients by luminance contours, but achieves this through excitatory modulation, moving from an abstract multiplicative representation to a biologically grounded mechanism that suggests a potential neural pathway through which luminance contours may influence filling-in.

In a broader sense, our work bridges neuroscience and neuromorphic engineering by translating biologically inspired mechanisms of color perception into a unified spiking architecture, in which complex visual percepts emerge from local event-based activity and temporal integration. Our model demonstrates how unconventional visual data, such as that produced by event cameras, can be processed without reducing it to static frames, offering conceptual guidance for the development of principled computational tools for neuromorphic imaging. Furthermore, the illusory afterimage can be interpreted as an inference error of the computational framework: the mismatch between the reconstruction (percept) and the sensory signal (stimulus), which can be evaluated systematically through configurable model parameters. This property can be particularly useful for informing design considerations and exploring potential failure modes in bio-inspired systems. Notably, Cohen-Duwek et al. (2021) used SNN-based filling-in for image reconstruction from event-camera data, demonstrating the applicability of spiking filling-in mechanisms in event-based imaging, though their approach targeted static image reconstruction, using a feed-forward filling-in implementation.

While our model is biologically plausible, being based on spiking neural computations that realize biologically grounded functional units, it does not follow neurons' detailed biophysical properties. For computational efficiency, most neural components are implemented using spiking rectified linear neurons, which lack some properties of real neurons, such as membrane leakage and refractory periods. However, this choice does not constitute a conceptual limitation of the model, and we expect the proposed architecture to be fully compatible with more biophysically aligned neuronal models, such as leaky integrate-and-fire (LIF) neurons, which are already used in the diffusion module. In fact, the rectified-linear response behavior implemented here can be well approximated using populations of LIF neurons.

The present work should also be distinguished from large-scale biologically detailed simulators such as Virtual Retina (Wohrer and Kornprobst, 2009). While both approaches involve spiking representations and aim for biological plausibility, they address fundamentally different modeling objectives and operate at different levels of abstraction. Virtual Retina focuses on the detailed simulation of retinal processing and on the generation of realistic spike trains that encode the visual stimulus, whereas our model accounts for perceptual effects, with a particular focus on the spatiotemporal dynamics of contour-driven filling-in and afterimage formation within an end-to-end spiking architecture.

The proposed model is limited to visual processing within a retinotopic reference frame without an internal mechanism for generating percepts at different spatial scales. As a consequence, it cannot account for multisensory phenomena such as the Taylor illusion (Taylor, 1941), in which an afterimage perceived on a hand moving in the dark changes its apparent size as a function of the proprioceptively inferred distance of the hand. At the same time, although modulatory signals in the present model are limited to stimulus-driven interactions, the model could be extended to incorporate external spatially selective modulatory signals through additional connections, without requiring changes to the core architecture, allowing future work to investigate the effects of top-down processes such as attention and awareness on afterimage formation (Bachmann and Murd, 2010; van Boxtel et al., 2010).

Although the overall simulation timeline generally aligns with the perceptual time course (Figures 5A, B), the build-up of filling-in is unrealistically slow. Experimental studies show that filling-in involves neural activity at multiple levels (Hong and Tong, 2017; Devinck and Knoblauch, 2019). However, our model uses a single filling-in layer, which limits each neuron's effective receptive field, and a simple four-neighbor scheme for horizontal connections. Together, these simplifications likely underlie the slow spread of color.

Nevertheless, the evolving progression of filling-in may provide relevant insights, particularly for the negative configuration. In this case, the contour interior comprises two distinct zones: a contour-adjacent region that was exposed to the chromatic inducer and a central region that was not. Given that luminance contours are known to enhance afterimages, percepts across these zones cannot be assumed to be identical. This potential difference should be considered when designing experiments and interpreting filling-in within the unexposed region. Hazenberg and van Lier (2013) reported that the perceived color is stronger in the negative condition. We suspect this difference may be driven, at least in part, by a perceptual bias toward the stronger afterimage in the chromatically adapted region, rather than reflecting stronger filling-in in the non-adapted interior. Given that participants in the original study viewed at least three cycles (6 seconds) with no upper limit, it is plausible that such an afterimage could have developed. Our model likewise predicts an enhanced contour-adjacent afterimage: under the same adaptation duration, the afterimage with a test contour is markedly stronger than in the null condition (Figure 4A). Furthermore, our model clearly demonstrates this bias: the average predicted color over the test contour interior differs depending on whether the adapted region is included. When the adapted region is excluded, the predicted chroma for the negative condition is similar to or weaker than that of the positive condition; including it consistently yields a stronger prediction for the negative condition (Figures 4E, 5B).

Our informal observations tentatively suggest that, under the negative condition, a stronger afterimage in the contour-adjacent region is not uncommon. Moreover, whereas the positive condition consistently yielded a uniform percept, the negative condition appeared more variable: a stronger afterimage could be accompanied by weaker or no filling-in; sometimes it even appeared opposite in hue; in other cases, uniform filling-in could be perceived. Naturally, systematic experiments are required to substantiate these tentative impressions. It also remains possible that the experimental conditions in the original study differed from ours, such that an enhanced afterimage was not a factor in their results. Still, variability across observers and stimulus colors should not be surprising; in fact, the findings reported by Hazenberg and van Lier (2013) already demonstrate significant variability. The uniform perception in the star stimulus may seem at odds with our concerns. However, in that case, the filling-in arises from a combination of both positive and negative effects, rather than from negative effects alone. In addition, there are marked geometric differences between the two stimuli, which may influence perception even if the underlying mechanisms are shared.

Interestingly, our model predictions (Figures 5A, B) progress through a sequence of perceptual variations that encompass the range of our tentative observations. This could potentially indicate that these variations arise from a common underlying mechanism, modulated by additional processes, that ultimately determines the resulting percept. Such modulation could be dependent on specific stimulus conditions, as well as reflect individual differences. Moreover, in an ideal diffusion process, the presence of the inner adapted edge (not amplified by the test contour) causes weaker filling-in inside it than in the adapted region. It is therefore possible that the strength of this adapted gradient determines the perceived filling-in: if the gradient is sufficiently weak, filling-in spreads unhindered, resulting in a uniform percept; if it is stronger, it may impede spreading, partially or completely, yielding distinct percepts across the two areas.

## Data Availability

All datasets generated for this study are included in the manuscript. The code supporting the conclusions of this article is available at https://github.com/NBELab/afterimage.

## References

[B1] Anstis S. Rogers B. Henry J. (1978). Interactions between simultaneous contrast and coloured afterimages. Vision Res. 18, 899–911. doi: 10.1016/0042-6989(78)90016-0 706164

[B2] Anstis S. Vergeer M. van Lier R. (2012). Luminance contours can gate afterimage colors and “real” colors. J. Vis. 12:2. doi: 10.1167/12.10.2 22961219

[B3] Bachmann T. Murd C. (2010). Covert spatial attention in search for the location of a color-afterimage patch speeds up its decay from awareness: Introducing a method useful for the study of neural correlates of visual awareness. Vision Res. 50, 1048–1053. doi: 10.1016/j.visres.2010.03.013 20347858

[B4] Barkan Y. Spitzer H. (2017). “The color dove illusion: chromatic filling-in effect following a spatial-temporal edge,” in The Oxford Compendium of Visual Illusions (Oxford University Press), 752–755. doi: 10.1093/acprof:oso/9780199794607.003.0109

[B5] Bekolay T. Bergstra J. Hunsberger E. Dewolf T. Stewart T. Rasmussen D. . (2014). Nengo: a python tool for building large-scale functional brain models. Front. Neuroinform. 7:48. doi: 10.3389/fninf.2013.00048 24431999 PMC3880998

[B6] Benda J. (2021). Neural adaptation. Curr. Biol. 31, R110–R116. doi: 10.1016/j.cub.2020.11.054 33561404

[B7] Cohen-Duwek H. Shalumov A. Tsur E. E. (2021). “Image reconstruction from neuromorphic event cameras using Laplacian-prediction and Poisson integration with spiking and artificial neural networks,” in 2021 IEEE/CVF Conference on Computer Vision and Pattern Recognition Workshops (CVPRW), 1333–1341. doi: 10.1109/CVPRW53098.2021.00147

[B8] Cohen-Duwek H. Slovin H. Ezra E. (2022). Computational modeling of color perception with biologically plausible spiking neural networks. PLoS Comput. Biol. 18:e1010648. doi: 10.1371/journal.pcbi.1010648 36301992 PMC9642903

[B9] Cohen-Duwek H. Spitzer H. (2018). A model for a filling-in process triggered by edges predicts “conflicting” afterimage effects. Front. Neurosci. 12:559. doi: 10.3389/fnins.2018.00559 30174580 PMC6107801

[B10] Cohen-Duwek H. Tsur E. E. (2021). “Biologically plausible spiking neural networks for perceptual filling-in,” in Proceedings of the Annual Meeting of the Cognitive Science Society, 43.

[B11] Cohen-Duwek H. Tsur E. E. (2022). “Biologically plausible illusionary contrast perception with spiking neural networks,” in 2022 IEEE International Conference on Image Processing (ICIP) (IEEE), 1586–1590. doi: 10.1109/ICIP46576.2022.9897264

[B12] Daw N. (1962). Why after-images are not seen in normal circumstances. Nature 196, 1143–1145. doi: 10.1038/1961143a0 14025557

[B13] Devinck F. Knoblauch K. (2019). Central mechanisms of perceptual filling-in. Curr Opin Behav Sci. 30, 135–140. doi: 10.1016/j.cobeha.2019.08.003

[B14] Eliasmith C. Anderson C. H. (2003). Neural Engineering: Computation, Representation, and Dynamics in Neurobiological Systems. London: MIT Press.

[B15] Francis G. (2010). Modeling filling-in of afterimages. Attent. Percept. Psychophys. 72, 19–22. doi: 10.3758/APP.72.1.19 20045876

[B16] Francis G. Ericson J. (2004). Using afterimages to test neural mechanisms for perceptual filling-in. Neural Netw. 17, 737–752. doi: 10.1016/j.neunet.2004.01.007 15288895

[B17] Grossberg S. Mingolla E. (1985). Neural dynamics of perceptual grouping: textures, boundaries, and emergent segmentations. Percept. Psychophys. 38, 141–171. doi: 10.3758/BF03198851 4088806

[B18] Grossberg S. Todorovic D. (1988). Neural dynamics of 1-d and 2-d brightness perception: a unified model of classical and recent phenomena. Percept. Psychophys. 43, 241–277. doi: 10.3758/BF03207869 3347487

[B19] Hazenberg S. van Lier R. (2013). Afterimage watercolors: an exploration of contour-based afterimage filling-in. Front. Psychol. 4:707. doi: 10.3389/fpsyg.2013.00707 24115940 PMC3792352

[B20] Hong S. W. Tong F. (2017). Neural representation of form-contingent color filling-in in the early visual cortex. J. Vis. 17:10. doi: 10.1167/17.13.10 29136409 PMC6097584

[B21] Kandel E. R. Koester J. D. Mack S. H. Siegelbaum S. A. (2021). Principles of Neural Science. Columbus, OH: McGraw Hill, 6 edition.

[B22] Kim J. Francis G. (2011). Color selection, color capture, and afterimage filling-in. J. Vis. 11, 1–15. doi: 10.1167/11.3.23 21454856

[B23] King W. L. Wertheimer M. (1963). Induced colors and colors produced by chromatic illumination may have similar physiological bases. Percept. Motor Skills, 17, 379–382. doi: 10.2466/pms.1963.17.2.379 14057248

[B24] Komatsu H. (2006). The neural mechanisms of perceptual filling-in. Nat. Rev. Neurosci. 7, 220–231. doi: 10.1038/nrn1869 16495943

[B25] McIlhagga W. Mullen K. (2018). Evidence for chromatic edge detectors in human vision using classification images. J. Vis. 18:8. doi: 10.1167/18.9.8 30208428

[B26] Pinna B. (2006). “The neon color spreading and the watercolor illusion: phenomenal links and neural mechanisms,” in Perceptual Organization and Visual Cognition, eds. J. Feldman, and M. A. Peterson (Cham: Springer), 235–254. doi: 10.1007/0-387-28898-8_17

[B27] Pinna B. Brelstaff G. Spillmann L. (2001). Surface color from boundaries: a new “watercolor” illusion. Vision Res. 41, 2669–2676. doi: 10.1016/S0042-6989(01)00105-5 11520512

[B28] Powell G. Bompas A. Sumner P. (2012). Making the incredible credible: afterimages are modulated by contextual edges more than real stimuli. J. Vis. 12:17. doi: 10.1167/12.10.17 23024354

[B29] Shapley R. Hawken M. J. (2011). Color in the cortex: single- and double-opponent cells. Vision Res. 51, 701–717. doi: 10.1016/j.visres.2011.02.012 21333672 PMC3121536

[B30] Shapley R. Núñez V. Gordon J. (2019). Cortical double-opponent cells and human color perception. Curr. Opin. Behav. Sci. 30, 1–7. doi: 10.1016/j.cobeha.2019.04.001

[B31] Shimojo S. Kamitani Y. Nishida S. (2001). Afterimage of perceptually filled-in surface. Science 293, 1677–1680. doi: 10.1126/science.1060161 11533495

[B32] Shively F. (1973). A new afterimage (colour contrast after-image?). Percept. Psychophys. 13, 525–526. doi: 10.3758/BF03205814

[B33] Spitzer H. Almon M. Sandler V. M. (1993). A model for detection of spatial and temporal edges by a single x cell. Vision Res. 33, 1871–1880. doi: 10.1016/0042-6989(93)90178-Y 8266643

[B34] St.Clair R. Hong S. W. Shevell S. (2007). Misbinding of color to form in afterimages. J. Vis. 7:366. doi: 10.1167/7.9.366 18321397

[B35] Taylor F. (1941). Change in size of the afterimage induced in total darkness. J. Exp. Psychol. 29, 75–80. doi: 10.1037/h0058125

[B36] Tsur E. E. (2021). Neuromorphic Engineering: The Scientist's, Algorithms Designer's and Computer Architect's Perspectives on Brain-Inspired Computing. New York: CRC Press. doi: 10.1201/9781003143499

[B37] van Boxtel J. J. A. Tsuchiya N. Koch C. (2010). Opposing effects of attention and consciousness on afterimages. Proc. Nat. Acad. Sci. 107, 8883–8888. doi: 10.1073/pnas.0913292107 20424112 PMC2889341

[B38] van de Sande K. E. A. Gevers T. Snoek C. G. M. (2010). Evaluating color descriptors for object and scene recognition. IEEE Trans. Pattern Anal. Mach. Intell. 32, 1582–1596. doi: 10.1109/TPAMI.2009.154 20634554

[B39] van Lier R. Vergeer M. (2009). Filling-in afterimage colors between the lines. Curr. Biol. 19, R323–R324. doi: 10.1016/j.cub.2009.03.010 19409278

[B40] von der Heydt R. Friedman H. S. Zhou H. (2003). Searching for the neural mechanism of color filling-in,” in *Filling-In: From Perceptual Completion to Cortical Reorganization* (Oxford University Press), 106–127. doi: 10.1093/acprof:oso/9780195140132.003.0006

[B41] Webster M. A. (2015). Visual adaptation. Ann. Rev. Vision Sci. 1, 547–567. doi: 10.1146/annurev-vision-082114-035509 PMC4742349 26858985

[B42] Williams D. R. Macleod D. I. (1979). Interchangeable backgrounds for cone afterimages. Vision Res. 19, 867–877. doi: 10.1016/0042-6989(79)90020-8 316228

[B43] Wohrer A. Kornprobst P. (2009). Virtual retina: A biological retina model and simulator, with contrast gain control. J. Comput. Neurosci. 26, 219–249. doi: 10.1007/s10827-008-0108-4 18670870

[B44] Zaidi Q. Ennis R. Cao D. Lee B. (2012). Neural locus of color afterimages. Curr. Biol. 22, 220–224. doi: 10.1016/j.cub.2011.12.021 22264612 PMC3562597

[B45] Zeki S. Cheadle S. Pepper J. Mylonas D. (2017). The constancy of colored after-images. Front. Hum. Neurosci. 11:229. doi: 10.3389/fnhum.2017.00229 28539878 PMC5423953

